# Supplementary material for: Impact of Lockdown on the Epidemic Dynamics of COVID-19 in France
Source: Front Med (Lausanne). 2020 Jun 5;7:274. doi: 10.3389/fmed.2020.00274 (PMC7290065; doi:10.3389/fmed.2020.00274)
Supplement: Supplementary file 2 [file Data_Sheet_2.pdf]

Supplementary Material

- The prior and marginal posterior distributions of the death rate γ are depicted in Fig. S1.
- The pairwise posterior distributions of the parameters (α, I_0), (α, γ), (α, κ), (γ, I_0), (γ, κ), (κ, I_0) are depicted in Fig. S2.
- To check the sensitivity of our results with respect to the choice of the initial number of susceptible individuals $S(t_0)$, we computed the values of R_e and $I(t_0)$ associated with the posterior mode, for several choices of $S(t_0)$. The results are depicted in Fig. S3.

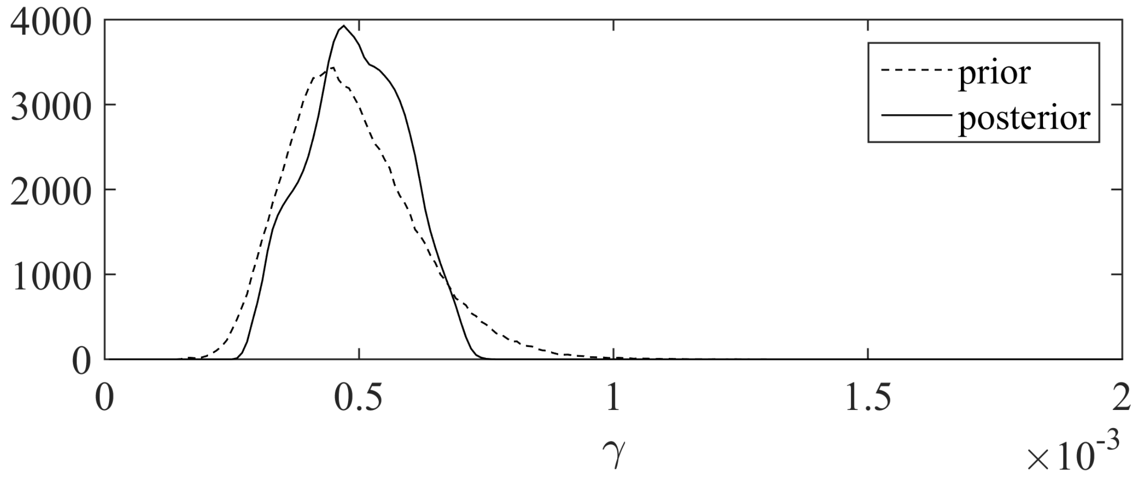

Figure S1. Prior and marginal posterior distributions of the death rate γ.

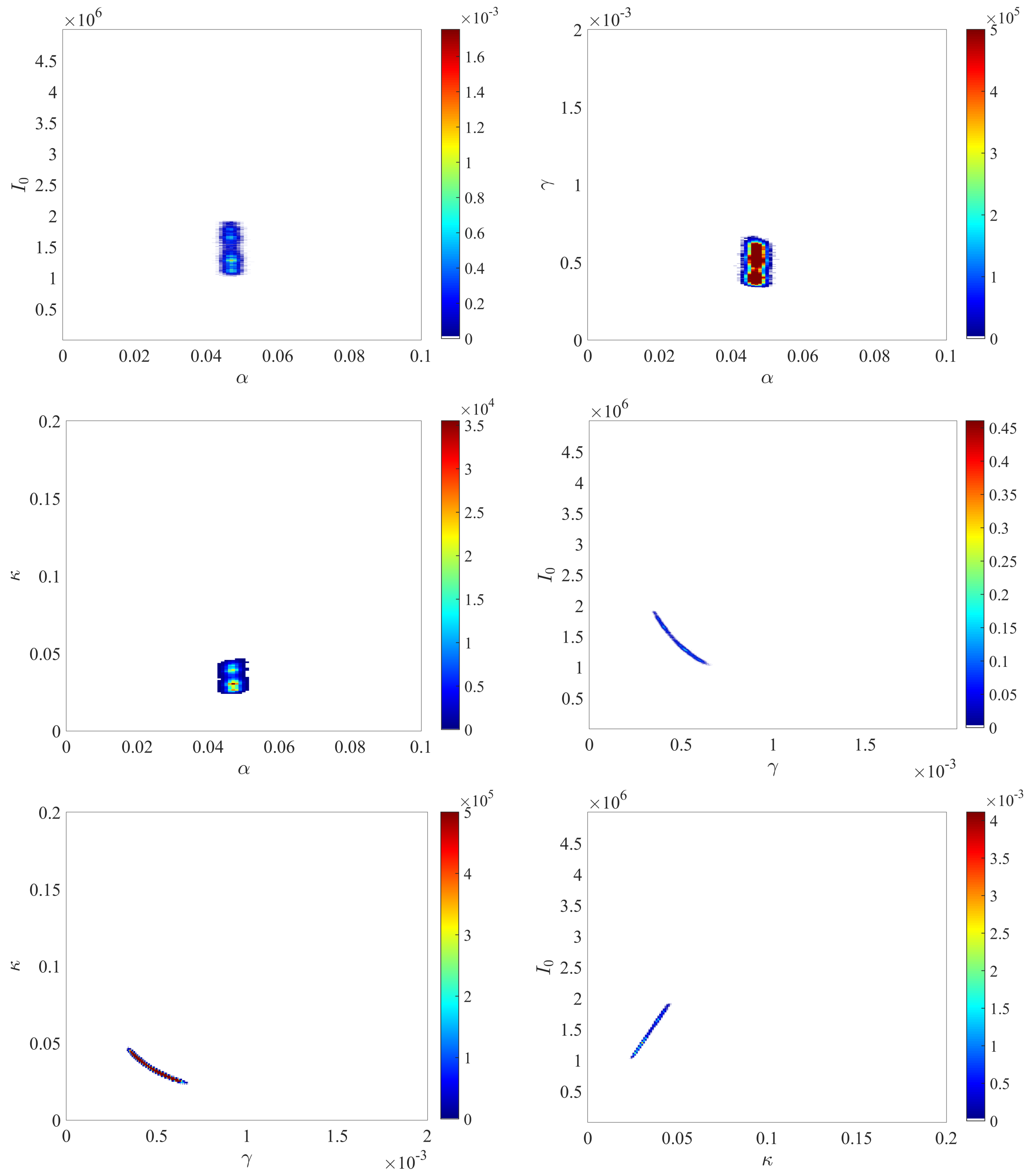

Figure S2. Joint posterior distributions of (α, I_0)**,** (α, γ)**,** (α, κ)**,** (γ, I_0)**,** (γ, κ) **and** (κ, I_0)**.**

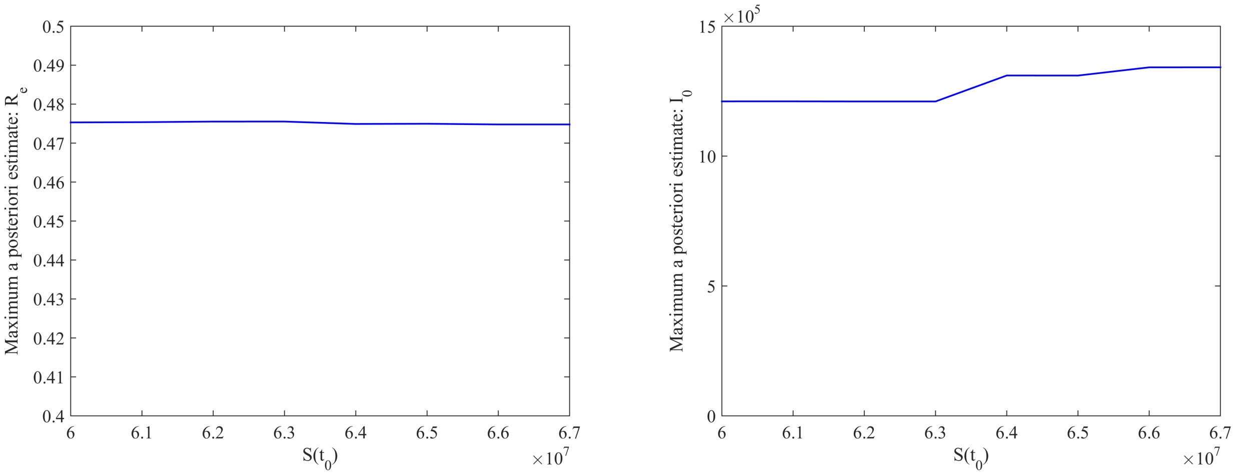

Figure S3. Dependence of the maximum posterior estimate of R_e and I_0 with respect to the initial number of susceptible individuals $S(t_0)$.
